# Searching for New Natural Compounds of Microbial Origin with Efficacy Against *Listeria monocytogenes* Biofilm Formation

**DOI:** 10.3390/foods15203704

**Published:** 2026-10-17

**Authors:** Mercedes de la Cruz, Sagrario Ortiz, Pilar Sánchez, Raquel Montiel, Jesús Martín, Ignacio González, Víctor González-Menéndez, José R. Tormo, Olga Genilloud, Francisca Vicente, Joaquín V. Martinez-Suárez, Juan L. Arqués

**Affiliations:** 1 Fundación MEDINA, Centro de Excelencia en Investigación de Medicamentos Innovadores en Andalucía, Parque Tecnológico de Ciencias de la Salud, Avenida del Conocimiento 34, 18016 Armilla, Granada, Spainpilar.sanchez@medinaandalucia.es (P.S.); jesus.martin@medinaandalucia.es (J.M.); ignacio.gonzalez@medinaandalucia.es (I.G.); victor.gonzalez@medinaandalucia.es (V.G.-M.); jose.tormo@medinaandalucia.es (J.R.T.); olga.genilloud@medinaandalucia.es (O.G.); francisca.vicente@medinaandalucia.es (F.V.); 2 Department of Food Technology, Instituto Nacional de Investigación y Tecnología Agraria y Alimentaria (INIA-CSIC), Carretera de La Coruña Km 7, 28040 Madrid, Spain; sortiz@inia.csic.es (S.O.); rakelm@inia.csic.es (R.M.); joaquin@inia.csic.es (J.V.M.-S.)

**Keywords:** natural compounds, microbial metabolite, high-throughput screening, *Listeria monocytogenes*, biofilms

## Abstract

*Listeria monocytogenes* causes listeriosis, a relatively rare but severe disease associated with high mortality rates, particularly among in high-risk populations. To reduce its presence in food production environments and increase the microbiological safety of food by identifying and developing new compounds that inhibit *L. monocytogenes* biofilm formation, this work joins the methodology of high-throughput screening (HTS) with the search for innovative applications of microbial natural products. Experimental conditions of an in vitro HTS assay have been set up to detect microbial extracts with antibiofilm-forming activity against a reference strain of *L. monocytogenes* (EGD-e, ATCC BAA-679) susceptible to disinfectants. Activity has been assessed by measuring fluorescence using resazurin (a redox indicator that allows quantifying the cell viability in biofilms). So far, 1520 microbial extracts have been tested, 127 of which have been selected based on their activity profile against the EGD-e strain. Subsequently, these extracts have been tested against a panel of five strains of *L. monocytogenes* resistant to disinfectants and persistently isolated from the meat industry, and their chromatographic profiles have been examined to prioritize their further study according to their activity and the presence of already known molecules. Twelve extracts have shown broad inhibition biofilm formation against the five resistant strains and three of them are candidates for further bioactivity-guided fractionation and structural elucidation. Further work is underway to characterize the chemical composition of these three extracts and explore their potential applications, with the aim of developing natural biocide formulations that may have a reduced environmental impact.

## 1. Introduction

*Listeria monocytogenes* is an opportunistic foodborne pathogen with specific virulence factors that enable it to cause listeriosis, a severe infection associated with high mortality rates in high-risk populations, such as older adults, neonates, pregnant women, and immunocompromised individuals [1,2,3]. *L. monocytogenes* can be found in different environments, such as water, soil, food products, humans, and animals [4,5]. As an environmental saprophyte, it is highly adapted to harsh conditions and competitive microbiota. The environments in which food is processed can promote its survival, as *L. monocytogenes* has the ability to form biofilms in the facilities and equipment of the food industry [3]. This increases the risk of food contamination and leads to critical food safety and public health issues, while having a significant economic impact. Bacterial cells associated with biofilms are more resistant to disinfectants than planktonic cells, making them difficult to remove and allowing them to persist in food production areas for long periods of time [6], demonstrating the importance of increasing the microbial safety of food through the discovery and development of new compounds with activity against *L. monocytogenes* biofilms [7,8].

Resistance to quaternary ammonium compounds in *L. monocytogenes* has been associated with the acquisition or expression of efflux-pump determinants, including *bcrABC* and *qacH*, although resistance, persistence, and biofilm formation should not be treated as interchangeable phenotypes [2,9]. The relationship between planktonic susceptibility, surface colonization, and survival in food-processing environments remains an important subject for investigation.

Microbial natural products represent a chemically diverse and valuable source of bioactive compounds for discovery and development. In this regard, Fundación MEDINA’s Natural Products Libraries have been built from one of the largest and most diverse microbial collections at the taxonomic, geographic, and environmental levels, covering a broad range of natural products’ chemical space and representing one of the major and most diverse sources to be used in the discovery of new bioactive compounds [10]. The potential of naturally derived compounds as promising candidates for the development of antimicrobials suggests their possible use as alternatives to conventional synthetic agents [11]. Thus, the aim of this work was to search for microbial extracts able to inhibit *L. monocytogenes* biofilm formation by establishing an high-throughput screening (HTS) approach to prioritize and identify microbial extracts that interfere with *L. monocytogenes* biofilm formation [12].

Herein we summarize the results of a screening approach that enabled the identification of three promising extracts as potential new microbial natural products with activity against *L. monocytogenes* biofilm formation.

## 2. Materials and Methods

### 2.1. Bacterial Strain

The *Listeria monocytogenes* EGD-e (ATCC BAA-679) reference strain [13] susceptible to disinfectants was used for the HTS experiments. Five BAC-resistant (BAC-r) *L. monocytogenes* belonging to serogroup IIa (S1, S10-1, S2-2, S2-3, and S10-3) isolated from a meat-processing plant [9] were used to assess the activity of the active extracts identified through the HTS assay.

Thawed stock inoculum suspensions were streaked onto tryptone soy agar (TSA, Biolife, Milano, Italy) plates and incubated at 37 °C for 24 h to obtain isolated colonies. Single colonies of this microorganism were selected and grown in 20 mL of tryptone soy broth (TSB, Biolife) in a 250 mL Erlenmeyer flask. The flask was incubated at 37 °C with shaking at 220 rpm for 24 h.

### 2.2. Fundación MEDINA’s Collection of Natural Products Extracts

Fundación MEDINA’s microbial collection holds 190,000 strains of filamentous fungi, actinomycetes and other bacteria from a wide range of geographical origins and with a great ecological and taxonomic diversity. The natural products library contains 140,000 extracts that were prepared from a selection of strains grown in multiple media and fermentation conditions. A small subset of the library comprising 1520 extracts enriched in secondary metabolites from both bacteria (880 extracts) and fungi (640 extracts) was tested against *L. monocytogenes* EGD-e. The selection of the extracts was based on their taxonomic diversity and the ecological relevance of the niches from which the strains were isolated. The 218 bacterial strains were isolated from soil, lichen, and plant rhizosphere samples collected in different arid environments of Granada and Almeria in Spain. The strains were assigned to 27 different genera, distributed across 15 families within the phylum *Actinomycetota*. Among them, 190 strains were identified at the species level by 16S rDNA sequencing. The 158 fungal strains were isolated from 42 rhizosphere samples from mountain fynbos and restios collected in Cape, Cederberg, Namaqualand, and Free State provinces of South Africa. The fungal isolates also represented a wide taxonomic diversity, with members of the orders *Diaporthales*, *Eurotiales*, *Helotiales*, *Hypocreales*, *Pleosporales*, and *Sordariales*. The high microbial diversity, for both bacteria and fungi, supported the chemical richness of the extract subset library, increasing the likelihood of identifying novel bioactive compounds.

Fungal strains were cultivated in LSFM medium (glycerol–glucose–yeast autolysate–soybean flour–tomato paste medium), MMK2 (mannitol–Murashige and Skoog salts medium, formulation 2), SMK-II (soluble-starch–maltose medium, formulation II), and YES (yeast extract–sucrose medium) [14]. Bacterial strains were grown in DNPM-2 (dextrin–N-Z Soy BL–primary yeast–MOPS medium, formulation 2), RAM-2, APM9, M016, SAM-6, and GPA production media [15,16], whose complete formulations are given in the cited protocols. Extracts were obtained using acetone (1:1) under agitation for 1 h in an orbital shaker. After extraction, samples were centrifuged at 1500× *g* for 15 min and the supernatant was concentrated to half of the original volume (2× whole broth equivalent, 2× WBE) in the presence of a final concentration of 20% dimethyl sulfoxide (DMSO, Sigma-Aldrich, St. Louis, MO, USA). Extracts were stored at −20 °C until needed [17].

### 2.3. Resazurin-Based Assessment of Biofilm Formation

The biofilm production assay on polystyrene microtiter plates was used to evaluate the ability of microbial extracts to prevent biofilm formation. The selected method to quantitatively measure the living cells present in the biofilm as an indirect metabolic readout was resazurin (RSZ)-based staining as previously described [18,19,20]. RSZ is a non-fluorescent blue dye which is converted by reduction reactions associated with respiration electron transferences (NADH, NADPH, FADH, or other biologically abundant reductive species in the presence of mitochondrial or cytoplasmic reductases) into resorufin, a pink highly fluorescent product. RSZ staining was performed as described by Paytubi et al. (2017) [21], with minor changes. Briefly, after removing the culture medium, the plates were washed twice with 200 µL of PBS 1× to remove non-adhering bacterial cells and dried with paper towels. Then, 100 µL of TSB, diluted 1:10 in distilled water and containing RSZ at 40 mg/L, was dispensed in each well and incubated in the darkn for three hours at 37 °C in static conditions. After that, data were quantified by measuring fluorescence (excitation 570 nm, emission 615 nm) using an EnVision™ Microplate Reader (Revvity, Inc., Waltham, MA, USA) and the activity of microbial extracts was expressed as the percentage by which RSZ was reduced after incubation time.

### 2.4. Biofilm HTS Assay

The experimental conditions of the in vitro HTS assay were optimized to identify microbial extracts that inhibit biofilm formation by the reference strain of *L. monocytogenes* EGD-e [20,21]. The HTS was performed in flat-bottomed and sterile 96-well polystyrene microtiter well plates (Greiner Bio-One, Frickenhausen, Germany) with the following layout: 10 µL of extract (2× WBE) was dispensed in each well using a Biomek^®^ FXP Automated Liquid Handler (Beckman Coulter Life Sciences, Indianapolis, IN, USA), and a volume of 100 µL of *L. monocytogenes* inoculum in TSB, previously adjusted from an overnight culture to a final concentration of 5 × 10^7^ CFU/mL, was added using a Multidrop™ Combi Reagent Dispenser (Thermo Fisher Scientific, Vantaa, Finland). Each assay plate contained control wells in the left and right columns distributed as follows: (1) negative controls (no biofilm generated), the four top wells of the left column containing 100 µL of TSB medium and 10 µL of 20% DMSO; (2) positive controls (total biofilm generated): the four bottom wells of the left column containing 100 µL of adjusted *L. monocytogenes* inoculum and 10 µL of 20% DMSO; (3) the eight wells of the right column including a dose–response curve (DRC) of benzalkonium chloride (BAC, Sigma-Aldrich) as reference control of biofilm development inhibitory activity [22], while ciprofloxacin (Sigma-Aldrich) was used as a negative control compound [23].

The assay plates were statically incubated at 37 °C for 24 h to allow biofilm formation. Total biomass (planktonic and biofilm) was monitored by determination of the OD_612nm_ before and after incubation using an EnVision™ Microplate Reader (Revvity). Staining with resazurin was performed as mentioned above, and the color shift was visually monitored. Blue-colored wells mean absence of metabolic activity (no biofilm generated or dead cells in the biofilm) while pink-colored wells mean cellular metabolic activity (biofilm formation or alive cells in the biofilm). Moreover, *L. monocytogenes* biofilms were quantified by measuring fluorescence (excitation 570 nm, emission 615 nm).

### 2.5. Evaluation of Inhibitory Activity Against Biofilm Formation and Statistical Data Analysis

Genedata Screener software (v18.0.4-Standard; Genedata, Inc., Basel, Switzerland) was used to process and analyze the fluorescence raw data imported directly from the EnVision™ Microplate Reader. The percentage of inhibition was expressed as a function of the percentage of resorufin fluorescence and was calculated using the normalization described by Paytubi et al. (2017) [21].

Quality control parameters, RZ’ factor and signal to background (S/B) ratio, were also determined. The Z′ factor describes the robustness of an assay by considering the mean and standard deviation of both positive and negative controls [24,25]. The robust Z′ factor (RZ′ factor) replaces the mean with the median and the standard deviation (SD) with the median absolute deviation. HTS experiments were performed in duplicate on different days (independent replicates) and extracts were selected as active when they displayed an average value ≥60% of inhibition of the biofilm formation assay.

### 2.6. Biofilm Assays Against Disinfectant-Resistant L. monocytogenes Strains

Five BAC-resistant (BAC-r) strains of *L. monocytogenes* (S1, S10-1, S2-2, S2-3, and S10-3) from a food production plant (meat industry) [9] were used to test them against the active extracts selected from the HTS assay. All the five strains were persistent contaminants in the environment of the food plant. These five BAC-r strains corresponding to five pulsed-field gel electrophoresis (PFGE) types were previously characterized by whole-genome sequencing (WGS) [26]. The S1 strain is a type ST31 low-virulence strain with mutations in the *inlA* and *prfA* genes and S10-1, S2-2, S2-3, and S10-3 strains are type ST121 strains which is one of the most prevalent STs worldwide and seems to be well adapted to the colonization of meat-associated environments [9,27]. The biofilm production assay on polystyrene microtiter plates was used to evaluate the ability of the extracts to prevent or reduce biofilm formation as described by Bonneville et al., 2020 [20]. The metabolic activities of *L. monocytogenes* biofilms were quantified in the absence or presence of the extracts. Two independent experiments were conducted (*n* = 16).

### 2.7. Liquid Chromatography (LC)–High-Resolution Mass Spectrometry (MS)

LC-MS analyses in low- (LR) or high-resolution (HR) mode were performed as previously described [28,29]. Chemical profiles of active extracts selected from the HTS assay were compared to Fundación MEDINA’s internal database of microbial metabolites. An in-house, constantly in development UV-MS database was used for dereplication with mass tolerance criteria of 0.002 Da and database matching using the predicted HR-MS molecular formula, retention time, and UV spectrum. When no matching in the database was observed, the predicted HR-MS molecular formula was manually searched in the commercial Chapman & Hall Dictionary of Natural Products (DNP v35.1) and public MS databases: Natural Products Atlas v.2024_09 and Lotus v.2022_06.

## 3. Results

### 3.1. Biofilm HTS Assay and Hit Selection

The reproducibility and the sensitivity of the biofilm HTS assay were assessed by determining susceptibility dose–response curves of two different antibacterial reference compounds (ciprofloxacin tested from 50 μg/mL to 0.098 μg/mL and BAC tested from 32 μg/mL to 0.06 μg/mL, respectively) against planktonic and biofilm growth of *L. monocytogenes* EGD-e, following CLSI guidelines [30] and the methodology mentioned above, respectively. The experiments were performed in triplicate. The MIC 90% (i.e., the minimum inhibitory concentration required to inhibit 90% of the growth of a microorganism) value obtained for both ciprofloxacin and BAC was 1.5 μg/mL and the MBIC 90% (i.e., the minimum concentration required to inhibit 90% of the formation of a biofilm) value for ciprofloxacin was >50 μg/mL and it was 16 μg/mL for BAC (Figure 1). MBIC/MIC ratios for both compounds were higher than 30- and 10-fold.

Additionally, the effect of DMSO on the biofilm growth of *L. monocytogenes* EGD-e was evaluated, as the microbial extracts to be tested in the HTS assay contained 20% DMSO. For this purpose, 12 different concentrations of DMSO ranging from 10 to 0.005% were tested, and the results showed that DMSO concentrations ≤1.82% had no effect on biofilm formation.

Upon biofilm assay validation, a subset of 1520 natural products (NPs) extracts were screened in duplicate at a single concentration (2× WBE), leading to a final assay concentration of 0.182 WBE and 1.82% DMSO.

RZ′ factors obtained were between 0.86 and 0.93 and S/B ratios were between 6.2 and 7.3, values that support the robustness and reproducibility of the HTS assays (Figure 2). Also, the MBIC value of BAC (16 μg/mL) was confirmed in every single assay plate, thus proving a consistent reproducible control within assay plates and between experiments performed on different days.

A total of 127 extracts (65 from 48 actinomycetes strains and 62 from 50 fungal strains) were selected based on their good antibiofilm-forming activity profile which was established as at least 60% inhibition on the biofilm formation assay against the EGD-e strain.

The 48 actinomycetes active strains were taxonomically identified as members of 12 different genera belonging to seven families, highlighting a remarkable diversity among the active taxa. These active strains were endophytes isolated from roots or various sources of soil samples, including plant rhizospheres. Regarding the 50 active fungal strains, only 15 of them were taxonomically identified and assigned to 11 different genera belonging to nine families and five orders, which also underscores the considerable diversity among the active fungal taxa.

### 3.2. Early LC-MS Dereplication

Dereplication by LC-MS analysis allows for fast selection of interesting extracts by detecting and identifying those containing potential new compounds. The 127 selected active extracts were subjected to dereplication against proprietary databases and 43 were shown to contain known compounds such as nocardamine, deferoxamine, rifamycin and phenazines, among others; 28 showed chromatographic profiles with very low signals; and 56 did not match any registered molecules either in Fundación MEDINA’s database of microbial metabolites or in the Chapman & Hall Dictionary of Natural Products (Table 1).

### 3.3. Biofilm Assays with Disinfectant-Resistant L. monocytogenes Strains

As a strategy to further characterize and prioritize the extracts according to their antibiofilm-forming activity, the 127 selected extracts were tested against a panel of five different BAC-r strains of *L. monocytogenes* (S1, S10-1, S2-2, S2-3, and S10-3) isolated from a food production plant (meat industry) [9].

Out of the 127 extracts tested, 78 did not display any activity on this panel of resistant strains while 49 exhibited inhibitory activity against biofilm formation of at least one of the five BAC-r strains (Table 1).

Within the 49 active extracts (30 from 27 actinomycetes strains and 19 from 14 fungi strains), 12 (five from actinomycetes and seven from fungi) showed inhibition of biofilm-forming ability against the whole panel of five BAC-r strains (Figure 3). The five actinobacteria corresponded to members of the families *Micromonosporaceae* (*Micromonospora vinacea*, *Pseudosporangium ferrugineum*, *Planctatinospora sonchi*), *Nocardiopsis umidischolae* and *Glycomyces sambucus*, all isolated from roots and rhizosphere samples in El Margen and Los Vados (Granada, Spain). Whereas all members of the family *Micromonosporaceae* presented very low signals when analyzed by LC-MS, and the activity could not be associated with any specific component. The siderophores deoxynocardamine (Terragine E) and nocardamine, both already shown to exhibit antibiofilm-forming activity on Gram positive pathogens, are produced by the other strains of *Nocardiopsis* and *Glycomyces* respectively (Table 2).

In the case of the seven fungal extracts, activities were produced by five strains isolated from the soil rhizospheres collected in South Africa of which three have been identified morphologically to the genus level as members of the genera *Pyrenochaetopsis*, *Clonostachys* and *Maroammaea*, the other two strains remaining as unidentified fungi. In the case of the two unidentified species, the cytotoxic compounds trichosetin, beauvericin, helvolic acid and sesquicillins were detected in the extracts. The strain of *Pyrenochaetopsis* was confirmed to produce phomasetin and altersetin, already reported with activity against Gram-positive bacteria among additional cytotoxic activities. Known molecules were not identified by LC-MS as produced by the strains of *Clonostachys* and *Maroammaea* (Table 2).

## 4. Discussion

Control of *L. monocytogenes* biofilms in food-processing environments remains a great concern and challenge as it is one of the main foodborne pathogens causing not only food-borne illness outbreaks but also important economic losses and product adulteration. Moreover, biofilm formation contributes to the persistence of this pathogen on food-contact surfaces and can increase its tolerance to antimicrobial interventions. This work aims to contribute to microbial safety of food through a primary screening workflow for prioritizing microbial extracts to prevent development of *L. monocytogenes* biofilms by merging the methodology of HTS with the search for new applications of microbial natural products. To our knowledge, this is the first study to combine high-throughput screening of a large microbial natural product extract collection with LC-MS dereplication and subsequent evaluation against a panel of benzalkonium-chloride-resistant *L. monocytogenes* strains isolated from a food-processing environment.

The results obtained during the setup of the *in vitro* HTS assay showed that the methodology was suitable for evaluating the effects of compounds on biofilm formation. Moreover, the reproducibility and sensitivity of the biofilm HTS assay were supported by the statistical values (RZ′ factor and S/B ratio) and the consistent BAC MIC value derived from all the experiments performed. Previous studies have demonstrated the suitability of HTS approaches for the identification of compounds with antibiofilm-forming activity. Nguyen et al. developed a validated HTS platform based on microbial natural product extracts and demonstrated its ability to identify compounds that inhibit biofilm formation or affect preformed biofilms [31]. This study also highlighted the importance of distinguishing antibiofilm-forming activity from conventional antimicrobial activity against planktonic cells. This distinction is particularly relevant to the present study, since our primary screening was specifically designed to identify microbial extracts that interfere with *L. monocytogenes* biofilm formation.

The MIC and MBIC values obtained for both ciprofloxacin and BAC were consistent with the substantially higher tolerance generally observed in biofilm-associated cells compared with planktonic cells and agreed with those reported for both compounds [2,23]. Previous studies have similar results that the antimicrobial susceptibility of *L. monocytogenes* differs between planktonic cells and biofilm-associated cells and depends on the antimicrobial compound and experimental conditions [32]. It is also important to highlight that the present HTS assay provides an efficient strategy for prioritization, and validation of the most promising extracts on food-contact materials will be an important next step.

From this screening approach, 127 microbial extracts were selected out of the 1520 screened as they showed antibiofilm-forming properties against the *L. monocytogenes* EGD-e strain. The subsequent LC-MS dereplication step provided an effective means of prioritizing these extracts according to their chemical profiles. The results obtained from the LC-MS analysis yielded 56 interesting extracts for which no registered molecules were detected in either Fundación MEDINA’s database of microbial metabolites or in the Chapman & Hall Dictionary of Natural Products (Table 1). These extracts were therefore prioritized for further investigation, although the absence of a database match does not constitute evidence of chemical novelty or biological activity.

Plant-derived compounds such as trans-cinnamaldehyde, carvacrol, thymol and eugenol have been shown to inhibit *L. monocytogenes* biofilm formation at subinhibitory concentrations, including on polystyrene and stainless-steel surfaces, indicating that natural products can affect biofilm-associated gene expression and interfere with biofilm development under conditions relevant to food-processing environments [8]. Similarly, cinnamaldehyde, eugenol, resveratrol and thymoquinone have been shown to inhibit *L. monocytogenes* biofilm formation in a concentration-dependent manner, with effects associated with changes in the expression of quorum-sensing genes [33]. These observations support the general concept underlying the present screening strategy, namely that natural products can provide chemically diverse molecules capable of interfering with *L. monocytogenes* biofilm development. However, most previous studies have evaluated defined plant-derived compounds, whereas the present approach explores microbial extracts as a source of structurally diverse secondary metabolites.

The use of disinfectant-resistant strains represents an additional feature of the present study. Forty-nine of the 127 selected extracts inhibited biofilm formation in at least one of the five BAC-resistant strains, and 12 extracts showed activity against the entire panel as well as the EGD-e strain (Figure 3). These results demonstrate that the primary screening step was effective in enriching for extracts with activity against multiple BAC-resistant strains. The two major types identified were the *prfA* mutant S1 (type ST31) and the strains containing Tn*6188* (type ST121). Both types were identified to be persistent contaminants in samples obtained from clean and disinfected surfaces of a RTE pork-product-producing plant [29].

The results of a comparative analysis of the five genomes also suggest that the two STs were equipped with different antimicrobial and stress resistance genes. The four ST121 strains contained Tn*6188* associated with the quaternary ammonium compounds resistance gene *qacH*, which may be related to a greater capability of the ST121 strains to survive in the presence of quaternary ammonium compounds (QACs) [9]. Additionally, the four ST121 *L. monocytogenes* strains harbored a plasmid that included the cadmium resistance transposon Tn*5422* and *clpL* genes, which most likely provide additional stress response potentials for the ST121 strains. A Tn*5422*-like transposon associated with the cadmium resistance gene *cadA1* was also found in the strain of PFGE type S1. In contrast, the particularly long persistence of ST31 could be related to the presence of a region identical to the so-called stress survival islet 1 (SSI-1). The five-gene SSI-1 can contribute to elevated resistance to several types of stress in certain strains of *L. monocytogenes* [9]. Therefore, the activity of selected extracts across this panel suggests that their antibiofilm effects are not restricted to a single disinfectant-resistant genetic background. Nevertheless, the present results do not establish whether the active extracts overcome the specific resistance mechanisms associated with BAC tolerance, and mechanistic studies will be required to determine the basis of their activity.

Interestingly, several of the active extracts contained metabolites with previously reported antibacterial activity. For example, altersetin, detected in one of the fungal extracts, is a fungal antibiotic with potent activity against several pathogenic Gram-positive bacteria [34]. Likewise, phomasetin, a fungal decalinoyltetramic acid metabolite, has also been reported to exhibit antibacterial activity against Gram-positive bacteria [35]. Among the 12 most interesting extracts exhibiting good antibiofilm-forming activity against both the five disinfectant-resistant strains and *L. monocytogenes* EGD-e, only three showed no database matches to known molecules under the conditions used. Two of these extracts were derived from fungal strains identified as *Mariannaea* sp. and *Clonostachys rosea* while the third from an actinomycete strain identified as *Micromonospora vinacea* (Table 2). Beyond their antimicrobial efficacy, these extracts may represent promising candidates of natural origin and could potentially provide more environmentally sustainable alternatives to conventional synthetic substances. Such potential environmental advantages, however, remain to be demonstrated and would require further assessment of their toxicity, biodegradability and ecological impact [11]. Of particular note, all three strains were isolated from rhizosphere samples as their ecological source, and their corresponding extracts are currently under investigation to purify and elucidate the structure of the active compounds. The detection of such metabolites in active extracts provides chemical support for the biological screening results and indicates that the workflow is capable of recovering extracts containing biologically relevant microbial secondary metabolites. However, because the present study evaluated crude extracts rather than purified compounds, the contribution of each individual metabolite to the observed antibiofilm phenotype cannot be established solely from the dereplication data. Synergistic or additive effects between co-occurring metabolites may also contribute to the activity.

Although the impact of these findings was not assessed in the present study, they represent a promising basis for the development of a natural and environmentally sustainable agent with potential applications in the food industry. This objective will guide our ongoing research.

## 5. Conclusions

The present study provided a robust screening tool with potential applicability in the biofilm research field and opens the possibility of performing screenings with a much larger collection of NPs, as our approach meets the demanding requirements of HTS drug discovery. Moreover, our project reinforces the notion that microbial products are an unlimited source of potential new antibacterial agents, offering promising alternatives for sustainable and effective control of bacterial contamination, especially in food industry applications.

## Figures and Tables

**Figure 1:**
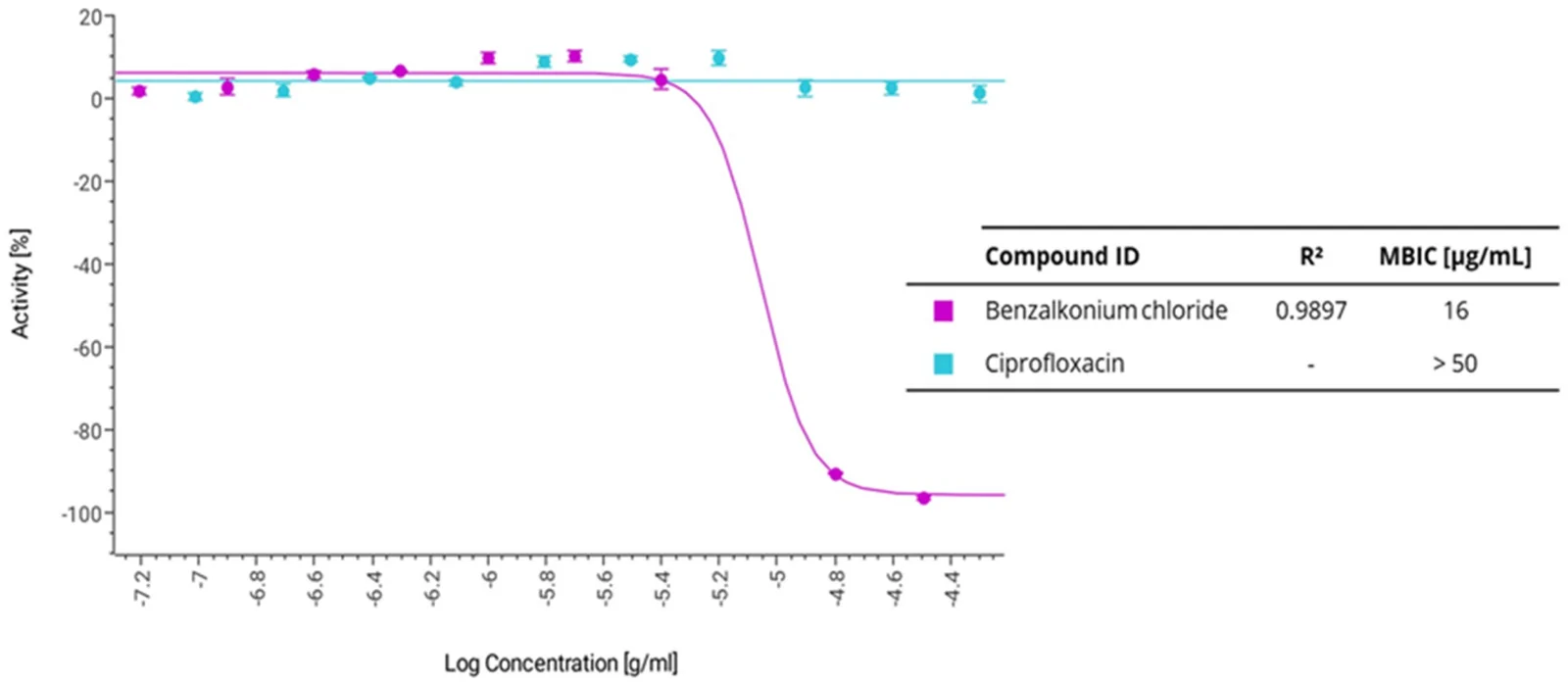
Antibiofilm-forming activity against *L. monocytogenes* EGD-e. Error bars indicate the SD of the mean (*n* = 6).

**Figure 2:**
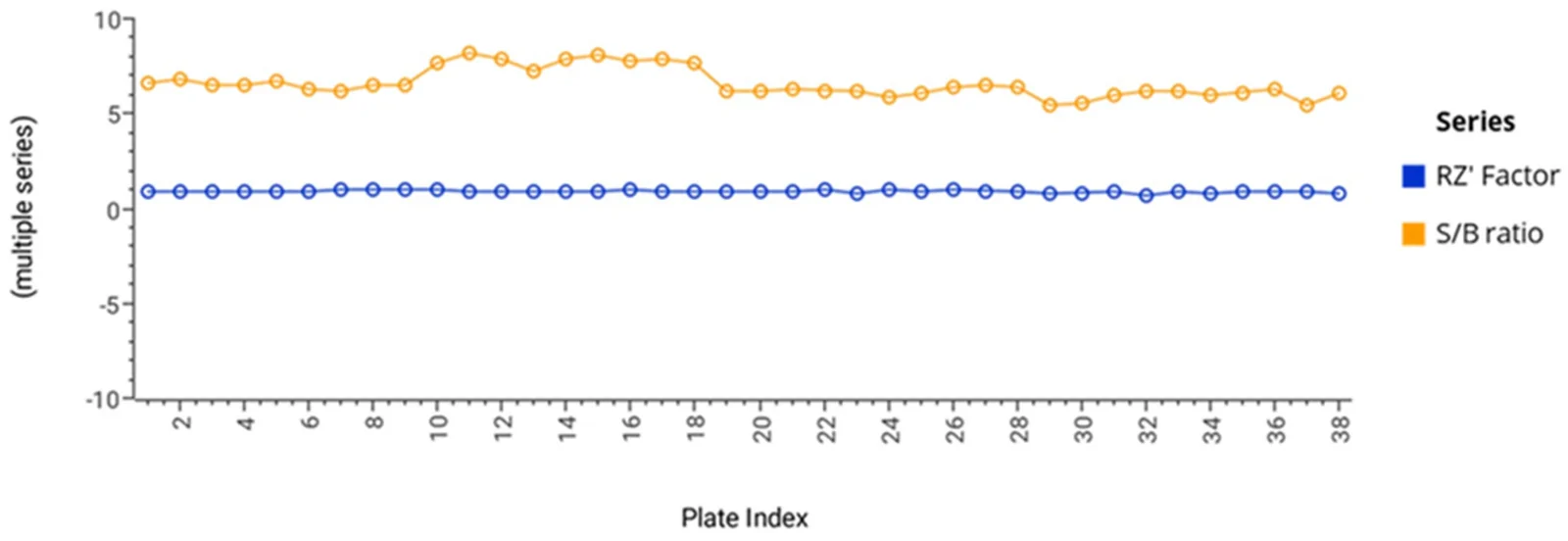
Robustness and reproducibility of the High-Throughput Screening (HTS) assays.

**Figure 3:**
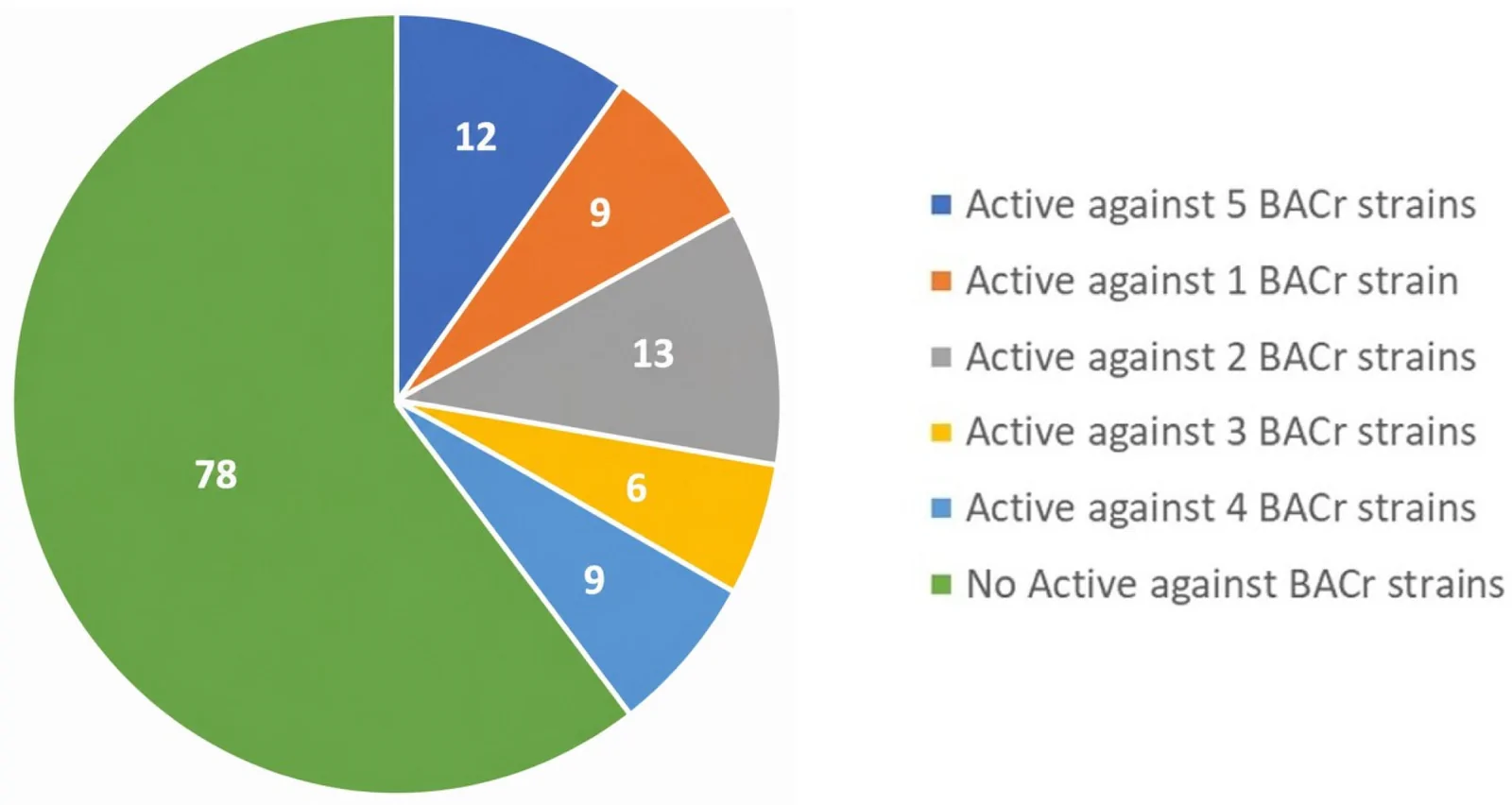
Antibiofilm-forming activity distribution of the 127 selected extracts against 5 benzalkonium-chloride-resistant (BAC-r) strains of *L. monocytogenes*.

**Table 1:** Biofilm-forming inhibition activity of the extracts against BAC-r strains of *L. monocytogenes* and Early Liquid Chromatography–High-Resolution Mass Spectrometry (LC-MS) dereplication.

N	Extracts	Early LC-MS Dereplication	BAC-r *L. monocytogenes* Strains					N	Extracts	Early LC-MS Dereplication	BAC-r *L. monocytogenes* Strains				
			S1	S10-1	S2-2	S2-3	S10-3				S1	S10-1	S2-2	S2-3	S10-3
1	CA-287907-a01-MR005-EC01-a01	-						65	CA-297347-a01-MR005-EC01-a01	-		+	+	+	
2	CA-288103-a01-MR005-EC01-a01	Desferrioxamine D1 + C_28_H_52_N_6_O_9_ (desferrioxamine no DNP)	+		+	+		66	CF-159395-a02-MR005-EC01-a01	Very low signals					
3	CA-289607-a03-MR005-EC01-a01	Terragine E	+	+	+	+	+	67	CF-159561-a02-MR005-EC01-a01	Infectopyrone					
4	CA-290195-a01-MR005-EC01-a01	-			+			68	CF-159561-a02-MR006-EC01-a01	Infectopyrone					
5	CA-290201-a01-MR005-EC01-a01	Deferoxamines		+				69	CF-159589-a02-MR005-EC01-a01	-					
6	CA-290202-a01-MR005-EC01-a01	-	+					70	CF-159607-a02-MR005-EC01-a01	Resorcylide					
7	CA-290206-a02-MR005-EC01-a01	6-Deoxy-8-O- -methylrabelomycin	+		+	+		71	CF-159616-a02-MR005-EC01-a01	-					
8	CA-290213-a01-MR005-EC01-a01	6-Deoxy-8-O- -methylrabelomycin)						72	CF-159637-a02-MR005-EC01-a01	Very low signals					
9	CA-290214-a01-MR005-EC01-a01	6-Deoxy-8-O- -methylrabelomycin)	+		+			73	CF-165303-a02-MR005-EC01-a01	Very low signals					
10	CA-290227-a01-MR005-EC01-a01	-	+	+	+	+		74	CF-165308-a02-MR005-EC01-a01	Dehydromassarilactone D					
11	CA-290229-a02-MR005-EC01-a01	6-Deoxy-8-O- -methylrabelomycin)						75	CF-165354-a02-MR005-EC01-a01	Talarazine B					
12	CA-290240-a02-MR005-EC01-a01	6-Deoxy-8-O- -methylrabelomycin)						76	CF-165401-a02-MR005-EC01-a01	Triacetylfusigen, Desferritriacetylfusigen					
13	CA-290242-a02-MR005-EC01-a01	6-Deoxy-8-O- -methylrabelomycin)	+			+		77	CF-165402-a02-MR005-EC01-a01	-		+	+	+	+
14	CA-290718-a03-MR005-EC01-a01	Very low signals						78	CF-165402-a02-MR006-EC01-a01	-					
15	CA-290885-a02-MR005-EC01-a01	Very low signals						79	CF-165405-a02-MR005-EC01-a01	Very low signals	+		+		
16	CA-290885-a02-MR006-EC01-a01	-						80	CF-165405-a02-MR006-EC01-a01	Roselipin 2A/2B, Glisoprenin D					
17	CA-291107-a02-MR005-EC01-a01	Very low signals	+	+		+	+	81	CF-165406-a02-MR005-EC01-a01	Very low signals					
18	CA-291107-a02-MR006-EC01-a01	Very low signals	+	+	+	+		82	CF-165844-a02-MR005-EC01-a01	-					
19	CA-291112-a01-MR005-EC01-a01	-		+		+	+	83	CF-165852-a02-MR005-EC01-a01	-					
20	CA-291118-a01-MR005-EC01-a01	Very low signals		+	+	+	+	84	CF-165858-a02-MR005-EC01-a01	-					
21	CA-291118-a01-MR006-EC01-a01	Very low signals	+	+	+	+	+	85	CF-165861-a02-MR005-EC01-a01	-					
22	CA-292817-a02-MR005-EC01-a01	Very low signals		+				86	CF-165863-a02-MR005-EC01-a01	-					
23	CA-292826-a02-MR005-EC01-a01	Very low signals						87	CF-165876-a02-MR005-EC01-a01	-					
24	CA-293028-a01-MR005-EC01-a01	Very low signals	+	+	+	+	+	88	CF-165879-a02-MR005-EC01-a01	-					
25	CA-293110-a01-MR005-EC01-a01	Deferoxamine		+	+	+	+	89	CF-165889-a02-MR005-EC01-a01	-	+				
26	CA-293320-a02-MR005-EC01-a01	Nocardamine	+	+	+	+		90	CF-165897-a02-MR005-EC01-a01	Very low signals					
27	CA-293320-a02-MR006-EC01-a01	-	+		+			91	CF-165899-a02-MR005-EC01-a01	-					
28	CA-293334-a01-MR005-EC01-a01	Deferoxamines	+			+	+	92	CF-165908-a02-MR005-EC01-a01	Talarazine B					
29	CA-293494-a02-MR005-EC01-a01	Nocardamine	+	+	+	+	+	93	CF-165919-a02-MR005-EC01-a01	-					
30	CA-293903-a02-MR009-EC01-a01	Very low signals						94	CF-165942-a02-MR005-EC01-a01	Trichosetin, Beauvericin	+	+	+	+	+
31	CA-294018-a02-MR009-EC01-a01	Very low signals						95	CF-165958-a02-MR005-EC01-a01	-	+			+	
32	CA-294018-a02-MR010-EC01-a01	-						96	CF-165964-a02-MR005-EC01-a01	Very low signals					
33	CA-294087-a01-MR005-EC01-a01	-	+	+	+	+	+	97	CF-165971-a02-MR005-EC01-a01	-					
34	CA-294906-a01-MR005-EC01-a01	Deferoxamines						98	CF-165974-a02-MR005-EC01-a01	-					
35	CA-296055-a02-MR005-EC01-a01	Very low signals	+			+		99	CF-166730-a02-MR005-EC01-a01	-					
36	CA-296065-a02-MR005-EC01-a01	Very low signals						100	CF-167624-a02-MR009-EC01-a01	-			+	+	
37	CA-296065-a02-MR006-EC01-a01	-						101	CF-167626-a02-MR005-EC01-a01	Helvolic acid, Sesquicillins	+	+	+	+	+
38	CA-296092-a02-MR005-EC01-a01	Collismycins						102	CF-167626-a02-MR006-EC01-a01	Helvolic acid	+	+	+	+	+
39	CA-296092-a02-MR006-EC01-a01	Collismycins						103	CF-167629-a02-MR005-EC01-a01	-					
40	CA-296100-a02-MR013-EC01-a01	Very low signals						104	CF-167631-a02-MR005-EC01-a01	Very low signals					
41	CA-296101-a02-MR005-EC01-a01	Rifamycins						105	CF-167638-a02-MR005-EC01-a01	-					
42	CA-296101-a02-MR006-EC01-a01	Rifamycins						106	CF-167645-a02-MR005-EC01-a01	-					
43	CA-296102-a02-MR005-EC01-a01	Very low signals						107	CF-167652-a02-MR005-EC01-a01	Very low signals			+		
44	CA-296104-a02-MR005-EC01-a01	-						108	CF-167652-a02-MR006-EC01-a01	-			+		
45	CA-296104-a02-MR006-EC01-a01	-		+	+	+	+	109	CF-167653-a02-MR005-EC01-a01	-					
46	CA-296109-a02-MR005-EC01-a01	-						110	CF-167663-a02-MR005-EC01-a01	Very low signals					
47	CA-296109-a02-MR006-EC01-a01	-						111	CF-167667-a02-MR005-EC01-a01	TAN 1786C, Glisoprenin D			+	+	
48	CA-296133-a02-MR005-EC01-a01	Very low signals						112	CF-167667-a02-MR006-EC01-a01	TAN 1786C, Glisoprenin D					
49	CA-296133-a02-MR006-EC01-a01	-	+			+		113	CF-167667-a02-MR007-EC01-a01	-					
50	CA-296143-a02-MR013-EC01-a01	-						114	CF-167669-a02-MR005-EC01-a01	-					
51	CA-297312-a01-MR005-EC01-a01	-						115	CF-167674-a02-MR005-EC01-a01	-					
52	CA-297312-a01-MR006-EC01-a01	-					+	116	CF-167674-a02-MR006-EC01-a01	Very low signals					
53	CA-297312-a01-MR007-EC01-a01	-						117	CF-167676-a02-MR005-EC01-a01	-					
54	CA-297313-a01-MR005-EC01-a01	-						118	CF-167676-a02-MR006-EC01-a01	-	+	+	+	+	+
55	CA-297313-a01-MR006-EC01-a01	-						119	CF-167681-a02-MR005-EC01-a01	SCH 60059, SCH 60065, SCH 60063			+		
56	CA-297315-a01-MR005-EC01-a01	Ferrioxamine E, Nocardamine						120	CF-167681-a02-MR006-EC01-a01	SCH 60059, SCH 60065, SCH 60063	+	+	+	+	
57	CA-297316-a01-MR005-EC01-a01	Terragine E, Nocardamine						121	CF-167685-a02-MR005-EC01-a01	-	+	+	+	+	+
58	CA-297316-a01-MR006-EC01-a01	Terragine E						122	CF-167689-a02-MR005-EC01-a01	Very low signals					
59	CA-297325-a01-MR005-EC01-a01	Terragine E, Nocardamine						123	CF-167690-a02-MR005-EC01-a01	-			+	+	
60	CA-297325-a01-MR006-EC01-a01	Terragine E						124	CF-167690-a02-MR006-EC01-a01	-			+	+	
61	CA-297330-a01-MR005-EC01-a01	-	+	+				125	CF-167722-a02-MR005-EC01-a01	Phomasetin	+	+	+	+	+
62	CA-297342-a01-MR005-EC01-a01	Arthrobactin		+	+	+		126	CF-167722-a02-MR006-EC01-a01	Altersetin, Phomasetin	+	+	+	+	+
63	CA-297343-a01-MR005-EC01-a01	Phenazines						127	CF-168005-a02-MR005-EC01-a01	3-hydroxy-5,7-dimethoxy-2-(2-oxopropyl)naphthalene-1,4-dione					
64	CA-297343-a01-MR006-EC01-a01	-	+		+										

DNP: Dictionary of Natural Products; (+) Reduced activity in the presence of extracts differed from controls without extracts (*p* < 0.05); Very low signals: compound levels were insufficient for reliable LC-MS dereplication.

**Table 2:** Taxonomic identification and origin of the bacterial and fungal strains whose active extracts exhibited strong antibiofilm-forming activity against the entire panel of five BAC-resistant (BAC-r) *L. monocytogenes* strains.

N	Extract Name	Early LC-MS Dereplication	Country Name ^1^	Subs Place ^2^	Source Type	Source Ecology ^3^	Phylum ^4^	Class ^5^	Tax Order ^6^	Family ^7^	Genus ^8^	Species ^9^
1	CA-289607-a03-MR005-EC01-a01	Terragine E	SP	LV	Plant	Root	Act	Actm	Stre	Noc	Noca	umi
2	CA-291118-a01-MR006-EC01-a01	Very low signals	SP	EM	Plant	Root	Act	Actm	Micr	Micro	Plan	son
3	CA-293028-a01-MR005-EC01-a01	Very low signals	SP	EM	Soil	Rhiz	Act	Actm	Micr	Micro	Pseu	fer
4	CA-293494-a02-MR005-EC01-a01	Nocardamine	SP	EM	Soil	Rhiz	Act	Actm	Glyc	Glyco	Glycom	sam
5	CA-294087-a01-MR005-EC01-a01	-	SP	EM	Soil	Rhiz	Act	Actm	Micr	Micro	Microm	vin
6	CF-165942-a02-MR005-EC01-a01	Trichosetin, Beauvericin	SA	CFS	Soil	Rhiz	ND	ND	ND	ND	UDI	fun
7	CF-167626-a02-MR005-EC01-a01	Helvolic acid, Sesquicillins	SA	NC	Soil	Rhiz	ND	ND	ND	ND	UDI	fun
8	CF-167626-a02-MR006-EC01-a01	Helvolic acid	SA	NC	Soil	Rhiz	ND	ND	ND	ND	UDI	fun
9	CF-167676-a02-MR006-EC01-a01	-	SA	MP	Soil	Rhiz	Asc	Sord	Hypo	Nect	Maro	sp.
10	CF-167685-a02-MR005-EC01-a01	-	SA	MP	Soil	Rhiz	Asc	Sord	Hypo	Bio	Clono	ros
11	CF-167722-a02-MR005-EC01-a01	Phomasetin	SA	FSP	Soil	Rhiz	Asc	Doth	Pleo	Cucu	Pyr	sp.
12	CF-167722-a02-MR006-EC01-a01	Altersetin, Phomasetin	SA	FSP	Soil	Rhiz	Asc	Doth	Pleo	Cucu	Pyr	sp.

^1^ SP: Spain; SA: South Africa. ^2^ LV: Los Vados (Granada); EM: El Margen (Granada); CFS: Central Free State; NC: Northern Cape; MP: Mpumalanga; FSP: Free State Province. ^3^ Rhiz: Rhizosphere. ^4^ Act: Actinomycetota; ND: Not determined; Asc: Ascomycota. ^5^ Actm: Actinomycetes; ND: Not determined; Sord: Sordariomycetes; Doth: Dothideomycetes. ^6^ Stre: Streptosporangiales; Micr: Micromonosporales; Glyc: Glycomycetales; ND: Not determined; Hypo: Hypocreomycetidaae; Pleo: Pleosporomycetidae. ^7^ Noc: Nocardiopsaceae; Micro: Micromonosporaceae; Glyco: Glycomycetaceae; ND: Not determined; Nect: Nectriaceae; Bio: Bionectriaceae; Cucu: Cucurbitariaceae. ^8^ Noca: Nocardiopsis; Plan: Plantactinospora; Pseu: Pseudosporangium; Glycom: Glycomyces; Microm: Micromonospora; UDI: Unidentified; Maro: Maroammaea; Clono: Clonostachys; Pyr: Pyrenochaetopsis. ^9^ umi: Umidischolae; son: Sonchi; fer: Ferrugineum; sam: Sambucus; vin: Vinacea; fun: Fungus; sp.: Species; ros: Rosea.

## Data Availability

The original contributions presented in this study are included in the article. Further inquiries can be directed to the corresponding author.
